# Beta‐casein in human milk is associated with infant stool frequency: A pilot proteomics study

**DOI:** 10.14814/phy2.71027

**Published:** 2026-08-05

**Authors:** Zhan‐Hua Li, Zhen‐Rong Xie, Meng Li, Jing‐Jing Xiong, Mei Liu, Yong‐Kun Huang

**Affiliations:** ^1^ Department of Pediatrics The First Affiliated Hospital of Kunming Medical University Kunming China; ^2^ The Medical Biobank The First Affiliated Hospital of Kunming Medical University Kunming China; ^3^ Yunnan Key Laboratory of Clinical Medicine Kunming China

**Keywords:** Beta‐casein, gut health, human milk, infant nutrition, infant stool frequency, milk proteins, proteomics

## Abstract

Breast milk proteins may regulate gut function and defecation frequency; however, their specific roles in these processes remain poorly understood. We hypothesized that variations in specific human milk proteins are associated with differences in stool frequency among exclusively breastfed infants. Infants were categorized into three groups based on defecation frequency: Group A (normal, 1–3 times per day), Group B (frequent, >3 times per day), and Group C (infrequent, ≤1 time every 3–4 days). Each group consisted of three biological replicates (total *N* = 9). This study was an exploratory pilot study. Breast‐milk samples were analyzed using tandem mass tag (TMT)‐based proteomics to identify differentially expressed proteins across groups. Gene Ontology (GO), KEGG pathway enrichment, and protein–protein interaction (PPI) analyses were conducted to explore functional pathways. A total of 2563 proteins were identified; β‐casein, α_s1_‐casein, and κ‐casein were significantly downregulated in infants with lower defecation frequency (Group C vs. Group A; fold change <0.6, *p* < 0.05). β‐casein showed the most significant difference (*p* = 0.0003). Enrichment analysis revealed pathways related to glycolipid metabolism, fibroblast proliferation, and coagulation cascades, while PPI analysis identified fibrinogen‐related proteins as key interaction nodes. These findings suggest that β‐casein levels in breast milk are associated with infant stool frequency. This may help clarify the association between constipation, diarrhea, and breastfeeding in infants and young children and may provide a theoretical reference for the subsequent development of targeted breast milk fortifiers and improvements in the intestinal environment in infants.

## 1 INTRODUCTION

Infant stool frequency is strongly influenced by feeding method; breastfed infants generally defecate more frequently than formula‐fed infants. However, considerable variability exists even among breastfed infants, with defecation frequencies ranging from as many as seven times per day to as few as once every 7 days (Moretti et al., 2019; Steurbauta et al., 2023). This wide range reflects the diverse nature of normal intestinal function in healthy infants. During the first year of life, both stool frequency and consistency undergo substantial changes, which can create considerable uncertainty for parents. Concerns about infant stool frequency and associated symptoms are among the most common reasons for pediatric consultations (Gatzinsky et al., 2023).

Despite their prevalence, infant defecation disorders are often inadequately managed, potentially leading to the development of functional gastrointestinal disorders (FGIDs). The Rome IV diagnostic criteria for FGIDs—an evolution of the Rome III criteria—define these conditions as disorders of brain–gut interaction characterized by (1) dysregulated gastrointestinal motility patterns, (2) visceral hypersensitivity, (3) altered mucosal immune function, (4) gut microbial dysbiosis, and (5) impaired central nervous system regulation of gut function (Geng et al., 2017). These disorders are highly prevalent in infants and young children, with an estimated global prevalence of 30% (Meng et al., 2023a). According to the Rome IV criteria, the prevalence of FGIDs in Chinese infants and young children is approximately 27.3%, with functional constipation being the most commonly reported issue (Huang et al., 2021). While previous research has explored various factors influencing infant gut health, including feeding methods, gut microbiota, and genetic predispositions, the specific role of breast milk proteins in regulating defecation frequency remains poorly understood. Breast milk contains numerous bioactive proteins that can influence intestinal function. However, their contribution to infant stool frequency and their potential role in the pathogenesis of FGIDs remain unclear.

Notably, human milk oligosaccharides (HMOs) and gut microbiota are central determinants of infant gut health. As key bioactive components in the early‐life gut ecosystem, they collectively modulate the intestinal microenvironment, promote immune maturation, and defend against pathogenic colonization. Their synergistic interaction supports gastrointestinal homeostasis, and accumulating evidence links this crosstalk to a reduced incidence of early‐life diarrhea. Therefore, it is essential to integrate breast milk proteins into the HMO‐microbiota‐gut axis framework to fully elucidate their coordinated contributions to infant gut health.

Recent research has investigated multiple components of breast milk, including protein types and quantities, bioactive ingredients, beneficial bacteria, cellular content, antibiotic‐resistance genes, and mobile genetic elements (Bzikowska‐Jura et al., 2020; Skjerven et al., 2022). Among these components, protein composition varies widely between individuals (Liu et al., 2019), and there is growing recognition that these proteins may affect infant stool frequency by modulating the intestinal flora. Breast‐milk proteins are known to regulate the infant gut microbiome, shaping its development and function (Xi et al., 2023). β‐Casein, a key milk protein, exhibits antibacterial properties that inhibit the growth of pathogenic bacteria and may therefore contribute to gut health (Dingess et al., 2021). β‐Casein plays a multifaceted role in neonatal development, demonstrating (1) immunomodulatory properties that support neonatal immune responses, (2) regulatory effects on gastrointestinal microbiota composition, and (3) intestinal‐protective functions (Liu et al., 2022). Notably, milk‐derived antimicrobial peptide‐1 (MAMP‐1), an endogenous β‐casein‐derived peptide identified in preterm human milk, enhances intestinal barrier integrity by upregulating zonula occludens‐1 (ZO‐1) expression in murine models, showing both prophylactic and therapeutic potential against necrotizing enterocolitis (NEC) (Long et al., 2025). In addition, β‐casein‐derived bioactive peptides—for example, β‐casomorphin‐9 (BCM‐9) from A2 β‐casein—have been shown to promote the growth of beneficial microbes such as Bifidobacteria by acting as prebiotics or by modulating metabolic pathways (Meng et al., 2023b). Collectively, these mechanisms suggest that β‐casein could influence defecation frequency through integrative effects on immune regulation, barrier function, and microbial ecology.

For example, Bruck et al. demonstrated that infant formula enriched with bovine α‐lactalbumin and casein glycomacropeptide (CGMP) favorably modulates the intestinal‐microbiota composition. A polypeptide produced by in vitro hydrolysis of α‐lactalbumin stimulated the growth of bifidobacteria and was therefore described as a “probiotic‐like” peptide (Brück et al., 2006). Similarly, Mastromarino et al. reported a strong association between lactoferrin (LF) concentrations in infant feces and the fecal microbial profile, highlighting the critical role of breast‐milk components in gut colonization (Mastromarino et al., 2014). In addition, κ‐casein in breast milk has been shown to protect the intestinal barrier and appears to be important for the early colonization of beneficial microbes (Lalor & O'Neill, 2019). Despite these insights, the specific contribution of individual milk proteins, particularly β‐casein, to ‐infant stool frequency and intestinal health has not been rigorously assessed in hypothesis‐driven studies. Given the exploratory nature of the present pilot study, these findings should be interpreted as hypothesis‐generating, and further validation in larger cohorts with mechanistic designs is warranted.

Breast‐milk proteins have been extensively studied for decades because of their central role in infant nutrition and development. Advances in proteomics, peptideomics, and glycoproteinomics now provide powerful tools for characterizing their composition and function. Among these tools, mass‐spectrometry‐based proteomics is fundamental for differential protein‐abundance analysis using relative‐quantification methods. Isovoltage labeling strategies such as TMT enable simultaneous quantification of multiple samples through multiplexing. This technology is particularly advantageous for detecting a broad spectrum of proteins—including highly acidic, highly alkaline, and low‐abundance species—making TMT one of the most widely used quantitative‐proteomics approaches (Dayon et al., 2021; Dayon et al., 2022; Kelly et al., 2021). Nevertheless, TMT has been applied only sparingly in breast‐milk proteomics; for instance, Chen et al. used TMT labeling to compare colostrum protein expression between mothers with and without gestational hypothyroidism (G‐HypoT) (Chen et al., 2018). Similarly, Zhang et al. employed TMT labeling combined with Nano‐LCQ Exactive HF tandem mass spectrometry (MS/MS) to investigate how geographic region and ethnicity influence the breast‐milk serum proteome (Zhang et al., 2019). Likewise, Gabriel and colleagues used Prosit‐TMT analysis to identify differences in proteome composition between morning and evening breast‐milk samples (Gabriel et al., 2022). These studies demonstrate the potential of TMT‐based proteomics for characterizing breast‐milk protein composition, yet they have focused on specific contexts. Comprehensive studies that leverage TMT to elucidate the relationship between breast‐milk protein composition and infant health outcomes, such as defecation frequency, remain scarce.

In the present study, we use TMT‐based proteomics to analyze breast‐milk protein composition, with particular emphasis on β‐casein, to explore its correlation with the defecation frequency of exclusively breastfed infants. Although the role of breast milk in infant gut health is well documented, detailed investigations of the association between milk‐protein composition and stool frequency are still limited. Furthermore, the mechanistic links between specific milk proteins and the development of FGIDs have yet to be elucidated. By addressing these gaps, this study aims to provide new insights into the regulatory role of milk proteins in early‐life gut health and their implications for managing infant gastrointestinal health. As an exploratory pilot study with a limited sample size, the findings are intended to generate hypotheses for future validation rather than to draw definitive conclusions.

## 2 MATERIALS AND METHODS

### 2.1 Research subject

From August 2020 to August 2021, nine healthy exclusively breastfed infants under 6 months of age, together with their mothers, were recruited from the outpatient department of pediatrics and preventive healthcare at our hospital. The inclusion criteria ensured that none of the infants received supplementary food during the study period. Infants were categorized into three groups based on stool frequency: Group A (normal, 1–3 times per day), Group B (frequent, >3 times per day), and Group C (infrequent, ≤1 time every 3–4 days). Each group consisted of three biological replicates (total *N* = 9). This study was an exploratory pilot study. Clinical data for both mothers and infants were collected through structured questionnaires. Breast‐milk specimens were collected, processed, and stored following standardized protocols. All samples were packaged according to experimental requirements and stored at −80°C to preserve their integrity. Transport to the testing center was conducted under cold‐chain conditions to maintain consistent temperature control and sample stability.

This study was conducted in accordance with the Declaration of Helsinki. The study and experimental procedures were approved by the Ethics Committee of the First Affiliated Hospital of Kunming Medical University (Ethical Batch Number: 2020 Lunshen L No. 23). Written informed consent was obtained from the guardians of all infant participants.

### 2.2 Methods

#### 2.2.1 Collection and preservation of breast‐milk specimens

The study enrolled only mothers 0.5–6 months postpartum (45–180 days). All collections were timed to occur during the final 2–3 min of feeding or expression to ensure compositional consistency. After obtaining informed consent, posterior breast milk was collected from participating mothers using standardized hand‐expression techniques in strict accordance with the Chinese Nutrition Society Guidelines for Human Milk Sample Collection and Storage (2022). Specifically, the collection was performed in a closed, clean room to avoid contamination. Mothers were instructed to sit comfortably to ensure natural breast sagging; prior to collection, they cleaned and disinfected their hands, then wiped both breasts with gauze soaked in sterile saline. Breasts were gently massaged for 1–2 min until the nipples became slightly erect—this step facilitated milk flow while maintaining specimen integrity. For manual expression, the thumb and index finger were placed 2 cm outward from the base of the nipple, at the edge of the areola, with the remaining three fingers supporting the lower part of the breast. A “press‐squeeze‐relax” cycle was applied: first, gentle pressure was exerted toward the deep part of the breast, followed by squeezing toward the nipple, with each cycle repeated every 3–5 s.

To maintain proteomic integrity, samples were immediately aliquoted into sterile cryovials (to prevent repeated freezing–thawing) and flash‐frozen at −80°C within 30 min of collection. During transportation to the testing center, samples were maintained in a cold chain (temperature consistently monitored at ≤−70°C) to avoid degradation. The lactation stage (postpartum days) was cross‐verified using maternal self‐reports, feeding logs, and clinical records. The median lactation stage did not differ significantly among groups (Kruskal–Wallis test, *p* > 0.05), minimizing potential intergroup variability in milk composition.

#### 2.2.2 Specimen determination

##### Protein extraction and trypsin digestion

Breast‐milk samples were retrieved from the −80°C freezer and immediately supplemented with 1% protease inhibitors to prevent protein degradation. The samples were subjected to ultrasonic lysis to disrupt cellular structures and release proteins. Protein concentrations were determined using a Bicinchoninic Acid (BCA) Protein Assay Kit (Beyotime, Shanghai, China) with a Microplate Reader (Bio‐Rad, Hercules, USA) (to measure absorbance at 562 nm) to ensure consistency across samples. Equal amounts of protein from each sample were enzymatically treated, and an appropriate quantity of standard protein was added to facilitate comparability. To precipitate the proteins, a final concentration of 20% Trichloroacetic Acid (TCA) (Sigma‐Aldrich, St. Louis, USA) was added gradually while vortexing the solution, followed by incubation at 4°C for 2 h. The precipitate was collected by centrifugation at 4500*g* for 5 min, washed with precooled acetone, and recentrifuged 2–3 times to remove impurities. After drying (using a Constant Temperature Forced Air Drying Oven, YiHeng Instruments Co., Ltd., Shanghai, China), the precipitate was resuspended in 200 mM Triethylammonium Bicarbonate Buffer (Sigma‐Aldrich) buffer by ultrasonication. Proteins were digested with trypsin (Promega, Madison, USA) at a 1:50 (protease: protein, w/w) ratio and incubated overnight at 37°C. To reduce disulfide bonds, 5 mM dithiothreitol (DTT) was added, and the solution was incubated at 56°C for 30 min. Subsequently, 11 mM iodoacetamide (IAA) was added to alkylate cysteine residues, and the mixture was incubated in the dark for 15 min.

##### TMT labeling

###### Reagent preparation

The TMT10plex™ labeling reagent (Thermo Scientific™, Cat. No. 90406) was equilibrated by thawing at room temperature for ≥20 min after removal from −80°C storage. The reagent was then centrifuged at 10,000 × g for 3 min to ensure complete sedimentation.

###### Peptide sample processing

Peptide samples were thawed on ice and centrifuged at 12,000 × g for 3 min at 4°C. The resulting pellets were resuspended in labeling buffer, vortexed for 1 min, and centrifuged again under the same conditions. Supernatants were carefully transferred to fresh microcentrifuge tubes.

###### Labeling reagent activation

The TMT reagent was dissolved in HPLC‐grade acetonitrile (Thermo Fisher Scientific) with vortex mixing for 30 s, followed by brief centrifugation at 14,000 × g for 5 s to achieve complete dissolution.

###### Peptide labeling reaction

The activated TMT reagent was combined with the peptide solutions, mixed by vortexing, and then centrifuged briefly (14,000 × g, 5 s). The reaction mixture was incubated at 25°C for 2 h with constant gentle agitation (300 rpm).

###### Quality control and efficiency assessment

Labeling efficiency was assessed by drying 5 μL aliquots of labeled peptide by centrifugal evaporation, reconstituting them in 0.1% trifluoroacetic acid (TFA) to a final concentration of 0.1 μg μL^−1^, adjusting the pH to <3 with 10% TFA (2 μL), pooling equal volumes, and desalting 5 μg of peptide using a SPE Strata‐X 10 mg/1 mL cartridge (Phenomenex, Torrance, USA). LC–MS/MS analysis demonstrated a labeling efficiency of 98.14% (2692 of 2743 peptides). Final validation was performed using Strata‐X C18 fractionation followed by PRM confirmation of labeled peptides.

##### HPLC fractionation

Peptide fractionation was performed using high‐pH, reversed‐phase high‐performance liquid chromatography (HPLC) with an Agilent 300Extend C18 column (5 μm particle size, 4.6 mm inner diameter, and 250 mm length) on a Liquid Chromatograph (Agilent, Santa Clara, USA). Peptides were separated with a gradient elution of 8%–32% acetonitrile (pH 9) over 60 min. The separation yielded 60 fractions, which were subsequently combined into 14 fractions based on retention time to reduce sample complexity. The pooled fractions were then vacuum‐freeze‐dried for further analysis.

##### Liquid chromatography‐mass spectrometry (LC–MS/MS) analysis

Peptides were separated on an EASY‐nLC 1200 ultra‐high‐performance liquid chromatography (UHPLC) system. Mobile phase A consisted of an aqueous solution containing 0.1% formic acid (Thermo Fisher Scientific) and 2% acetonitrile (Thermo Fisher Scientific), whereas mobile phase B contained 0.1% formic acid and 90% acetonitrile (Thermo Fisher Scientific). The gradient elution program was as follows: 7%~23% B over 0–60 min, 23%~32% B over 60–82 min, 32%~80 % B over 82–86 min, and 80% B from 86 to 90 min. The flow rate was maintained at 500 nL/min throughout. Separated peptides were ionized via nanospray ionization (NSI) at 2.2 kV and analyzed on a Q Exactive™ HF‐X mass spectrometer. Primary MS scans were acquired over an m/z range of 350–1600 at a resolution of 120,000. The 20 most intense peptide ions in each survey scan were selected for higher‐energy collisional dissociation (HCD) with a normalized collision energy of 28%. Secondary MS scans were acquired over an m/z range starting at 100 at a resolution of 30,000. Automatic gain control (AGC) was set to 3 × 10^6^, with a maximum injection time of 50 ms. A dynamic exclusion window of 30 s was applied to prevent repeated analysis of the same precursor ion. The peptide identification and protein quantification in this proteomic analysis were performed using MaxQuant (v1.6.15.0), with the quantification method set to TMT‐10plex. Database search was performed with standardized mass tolerance parameters as follows: the primary precursor ion mass tolerance was set to 20 ppm for the initial search and optimized to 4.5 ppm for the main search; the secondary fragment ion mass tolerance was set to 20 ppm. For basic matching parameters, Trypsin/P enzyme digestion mode was adopted with a maximum of 2 missed cleavage sites. The minimum amino acid length of identified peptide segments was defined as 7, and the maximum number of modifications per peptide segment was limited to 5. The false discovery rate (FDR) was set to 1% for both peptide spectrum match (PSM) and protein identification.

##### Protein function annotation and quantitative analysis

The TMT‐10plex labeling quantitative method was used in this experiment. The core quantitative filtering and quality control standards were as follows: Modification filtering: Carbamidomethyl (C) was set as a fixed modification, while N‐terminal acetylation, methionine oxidation, and asparagine/glutamine deamidation (NQ) were set as variable modifications. Peptide admission criteria: Only peptide segments with a length of ≥7 amino acids were included in identification and quantification. Quality control admission: Only protein, peptide, and PSM results with FDR <1% were included in the final quantitative analysis.

The analysis of mass‐spectrometry data provides a signal intensity value (I) for each peptide segment across different samples. Based on this information, the relative quantification of proteins is calculated using the following steps: (i) First, I for each peptide segment across different samples is normalized. The relative quantitative value (R) for each peptide segment in each sample is calculated with the formula: Rij = Iij/Mean(Ij), where i represents the sample, and j denotes the peptide segment. (ii) Each relative quantitative value (Rij) is further adjusted through median normalization. The normalized relative value (NR) is obtained with NRij = Rij/Median(Ri). Finally, the relative quantitative value for a protein is determined as Rik = Median(NRij, j∈k), where k represents the protein, and j corresponds to peptide segments associated with that protein. This project involves enzymatic labeling, mixing, and machine operation within the same batch; therefore, batch effects were not considered. Technical variability was assessed using correlation and principal component analyses (PCAs).

##### Bioinformatics analysis and processing

Target proteins were annotated with the Gene Ontology (GO) and Kyoto Encyclopedia of Genes and Genomes (KEGG) databases via the ClusterProfiler R package. This procedure involved sequence comparison, GO‐term extraction, and supplementary annotation, covering biological processes, cellular components, and molecular functions. KEGG‐pathway analysis provided insights into metabolic and signaling pathways associated with the target protein set.

Enrichment analysis was conducted with Fisher's exact test, and significance levels were calculated as adjusted *p* values <0.05 for GO terms and KEGG pathways relative to the overall proteome. Results were visualized with bar plots and pathway maps. Cluster analysis was then performed on the enriched GO classifications, KEGG pathways, and protein domains across the comparison groups. Hierarchical clustering with Euclidean distance identified functional correlations among differentially expressed proteins, and the results were displayed as heat maps.

Differentially expressed proteins with a fold‐change threshold ≥1.3 were selected for protein–interaction analysis. Protein database identifiers or protein sequences were queried against the STRING (v 11.0) protein–protein interaction database. Networks were constructed with a confidence score >0.7, highlighting key interactions. Network properties, such as node degree and clustering coefficient, were analyzed to identify central hub proteins, which were further correlated with the GO and KEGG enrichment results.

### 2.3 Statistical methods

Statistical analysis was performed with SPSS version 20.0. Differentially expressed proteins were identified at a fold‐change threshold of ≥1.3 or ≤0.6 and *p* < 0.05, in accordance with commonly accepted standards in analogous proteomic studies (Wang et al., 2019). This threshold enables effective identification of proteins with significant expression differences while reducing false positive results. Normally distributed data are presented as mean ± standard deviation (x̄ ± s), and intergroup differences were evaluated with a two‐tailed *t*‐test. Non‐normally distributed data were analyzed with the Mann–Whitney *U* test. For multi‐group comparisons, one‐way ANOVA followed by Bonferroni post hoc correction was applied. Because of the small sample size (*n* = 3 per group), intergroup statistical tests for clinical characteristics were not performed. Instead, baseline homogeneity was assessed by comparing the numerical ranges of key variables (e.g., gestational age, birth weight, and maternal pre‐pregnancy BMI) to minimize potential confounding effects.

## 3 RESULTS

### 3.1 Clinical characteristics of mothers and infants

To ensure comparability, we examined baseline clinical data from mothers and infants to rule out confounding factors. The mean maternal age was 31.3 years, the mean prenatal BMI was 20.4 kg/m^2^, and the mean infant birth weight was 3174.4 g. Given the exploratory nature of this pilot study and its limited size (*N* = 3 per group), we performed descriptive rather than inferential analyses to evaluate baseline characteristics. The results showed comparable distributions across Groups A, B, and C for all measured parameters, including infant demographics (age, birth weight, and gestational age), maternal factors (age, pre‐pregnancy BMI), socioeconomic status (family income and maternal employment), and lifestyle variables (dietary habits, sleep quality, and psychological factors), as detailed in Tables 1 and 2. These findings confirmed essential homogeneity among the groups before the proteomic investigation.

**TABLE 1:** Clinical data on mothers and infants.

Group	A	B	C
Baby gender			
Male gender, *n* (%)	2 (66.67%)	2 (66.67%)	3 (100%)
Baby age (month)	4.17 ± 1.44	3.5 ± 1.32	4.17 ± 0.29
Birth weight (g)	3143.33 ± 290.23	3230 ± 60.83	3150 ± 50
Baby nationality			
Miao	0	1	0
Han	3	2	3
Parity			
1	2	3	1
2	1	0	2
Gestational weeks (w)	38.1 ± 1.57	39.81 ± 0.46	39.71 ± 0.38
Delivery mode			
Natural birth	3	2	3
Caesarean section	0	1	0
Mother age (years)	28.67 ± 4.04	31 ± 3.61	34.33 ± 1.53
Mother BMI	19.03 ± 0.65	20.93 ± 0.78	21.33 ± 1.86
Sleep quality			
3–4 h	1	0	1
5–6 h	1	0	2
7–9 h	0	3	0
≥10 h	1	0	0
Psychological factors			
Happy	2	2	2
Not so	1	1	0
Depressed	0	0	2
Work experience			
Continue to work until prenatal	1	3	2
Out of work	2	0	0
Not working in the third trimester	0	0	1
Family income			
3000–5000	0	1	0
5000–800	1	1	1
≥8000	2	1	2
Mother physical condition			
In good health, occasionally catch a cold	3	3	3
Breastfeeding frequency			
Every 2–3 h	1	2	0
Every 3–4 h	2	1	3
Each breastfeeding			
Both sides were vacuumed	1	1	0
One side was vacuumed, the other was not	2	1	3
Both sides were not vacuumed	0	1	0

**TABLE 2:** Mother's eating habits.

Group	A			B			C		
ID	19‐M‐1	24‐M‐1	27‐M‐1	34‐M‐1	35‐M‐1	70‐M‐1	37‐M‐1	38‐M‐1	50‐M‐1
Meat	Yes	Yes	Yes	Yes	Yes	Yes	Yes	Yes	Yes
Milk	Yes	Yes	Yes	Yes	Yes	Yes	Yes	Yes	Yes
Eggs	No	Yes	Yes	No	Yes	Yes	Yes	Yes	Yes
Beans	Yes	Yes	Yes	No	Yes	No	Yes	Yes	Yes
Vegetables	Yes	Yes	Yes	Yes	Yes	Yes	Yes	Yes	Yes
Rice and noodles	Yes	Yes	Yes	Yes	Yes	Yes	Yes	Yes	Yes

### 3.2 TMT detection results

**FIGURE 1:**
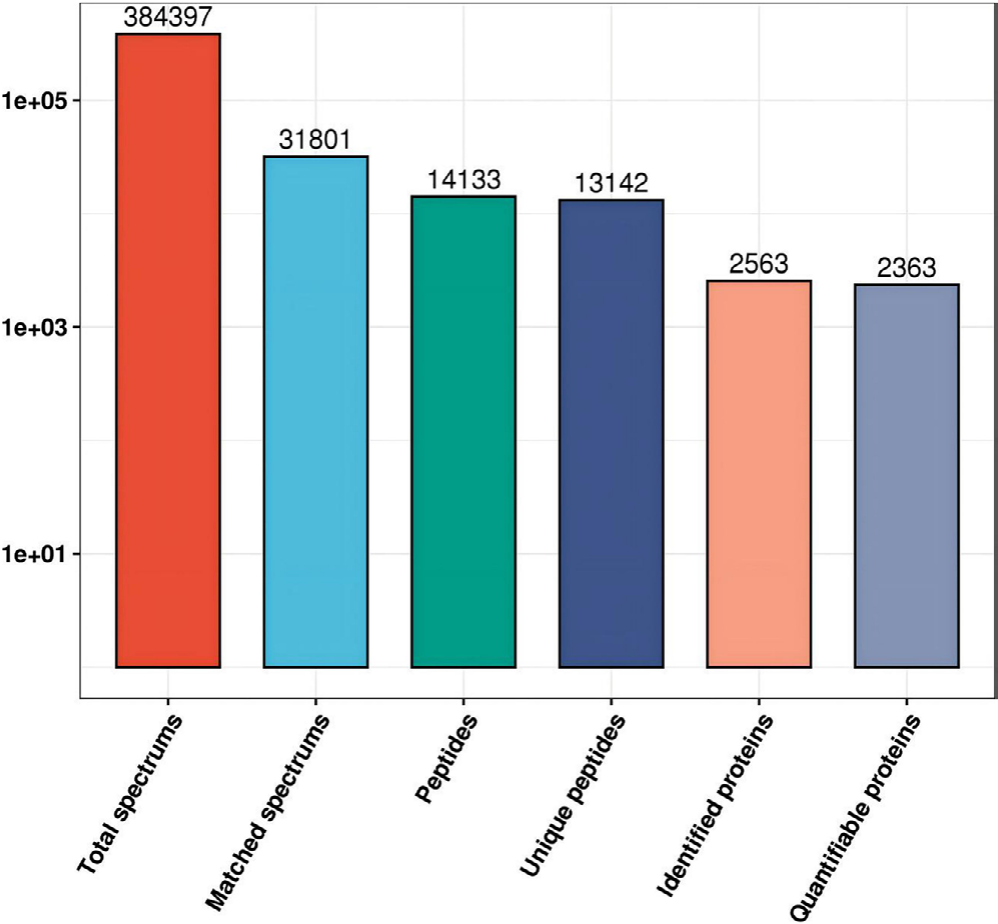
Overview of protein identification. Summary of protein‐identification results. A total of 2563 proteins were identified from breast‐milk samples with the Homo_sapiens_9606_SP_20201214.fasta database. Of these, 2363 proteins were quantified, providing the foundation for all subsequent differential‐expression analyses.

To analyze differences in breast‐milk protein composition among Groups A, B, and C, TMT‐based proteomics was performed. The analysis generated 384,397 spectra, of which 31,801 were usable, yielding 14,133 peptide segments. Among these, 13,142 were unique. In total, 2563 proteins were identified, and 2363 were reliably quantified (Figure 1). Protein identification relied on the Homo_sapiens_9606_SP_20201214.fasta database, a high‐quality human protein repository that ensures accurate identification and provides a solid foundation for downstream functional annotation and differential analysis.

During protein detection and quantification, the 31,801 effective spectra ensured high sensitivity and accuracy in peptide identification. The 13,142 unique peptides further confirmed the robustness of the database‐driven approach. Of the 2563 proteins detected, 2363 met stringent quantification criteria, yielding a comprehensive and reliable dataset for subsequent analyses. To minimize technical variability, TMT intensities were normalized with the median‐ratio method, and proteins displaying abnormal intensity values were excluded as outliers. Principal‐component analysis (PCA) of the normalized data revealed clear clustering of samples by Groups A, B, and C, indicating distinct proteomic signatures associated with defecation frequency.

### 3.3 Differential protein expression analysis

The TMT‐proteomics dataset—comprising 2563 identified proteins and 2363 quantified proteins—served as the basis for differential expression analysis. By comparing normalized protein intensities across Groups A, B, and C, we identified key proteins associated with variations in defecation frequency, offering insight into the molecular mechanisms underlying infant intestinal health. Between group C (reduced defecation) and group A (normal defecation), 381 proteins were differentially expressed, including 213 upregulated and 168 down‐regulated proteins. Between group B (increased defecation) and group A, 147 proteins were differentially expressed (94 are up‐modulated, 53 down‐modulated). Between group C and group B, 38 proteins were differentially expressed (18 are up‐modulated and 20 down‐modulated).

**FIGURE 2:**
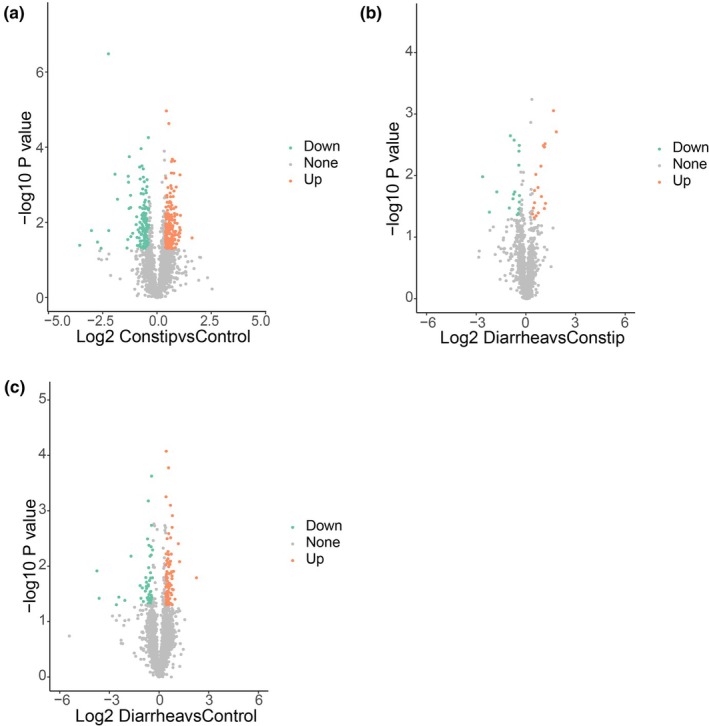
Volcano plot of differential protein expression. Volcanic plots showing differential protein expression across Group C (reduced defecation) versus Group A (a), Group B (increased defecation) versus Group A (b), and Group B versus Group (c). The *x*‐axis represents the log^2^‐transformed fold change of protein expression, and the *y*‐axis represents the −log^10^ *p* value of statistical significance. Orange dots indicate significantly differentially expressed proteins (*p* ≤ 0.05; fold‐change ≥1.3 or ≤1/1.3), while gray dots represent proteins without significant changes.

Volcano plot (Figure 2) illustrates the significant changes in protein expression across groups. FDR‐corrected *p* values for key differentially expressed proteins are provided in Table S1 for reference.

### 3.4 GO functional enrichment analysis

**FIGURE 3:**
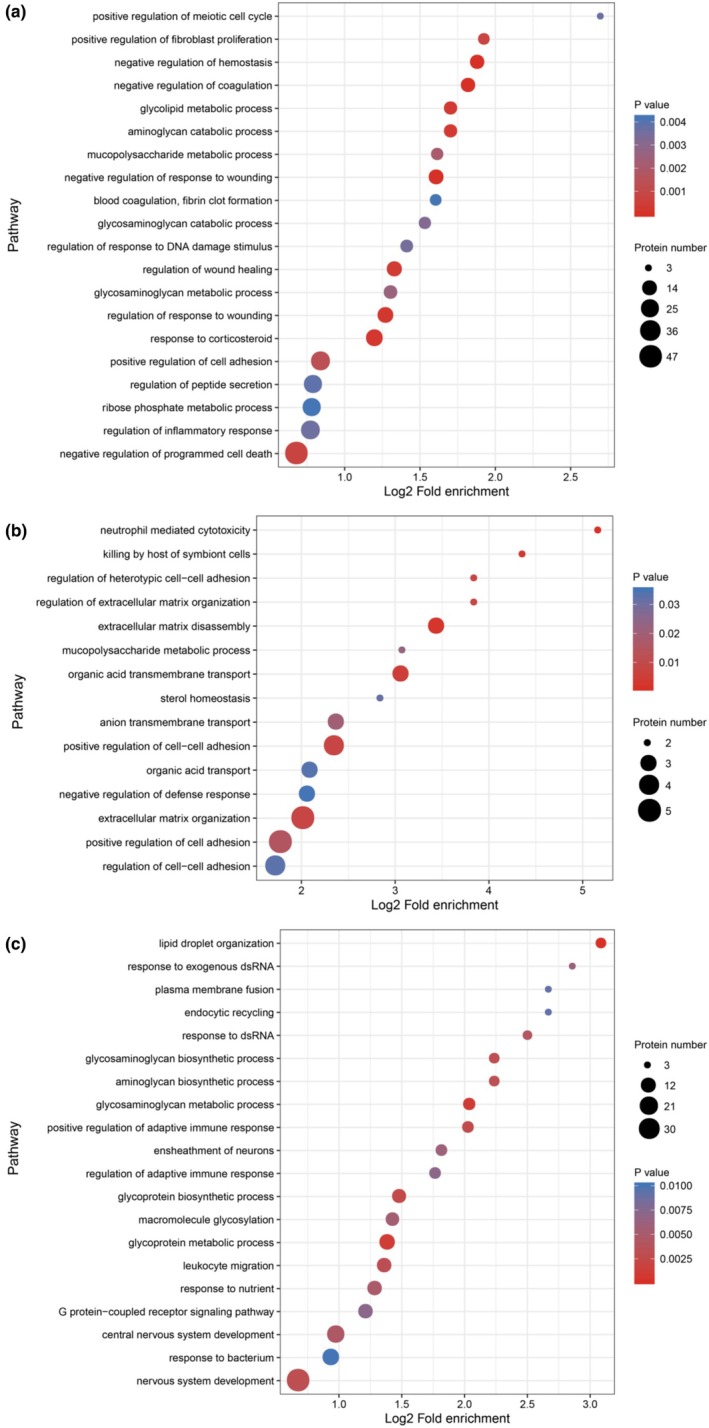
GO biological process (BP) enrichment bubble plots of differentially expressed proteins. (a) Group C versus Group A; (b) Group C versus Group B; (c) Group A versus Group B. Bubble color indicates enrichment significance (*p* value), with a blue‐to‐red gradient; redder bubbles correspond to smaller *p* values and higher statistical significance.

**FIGURE 4:**
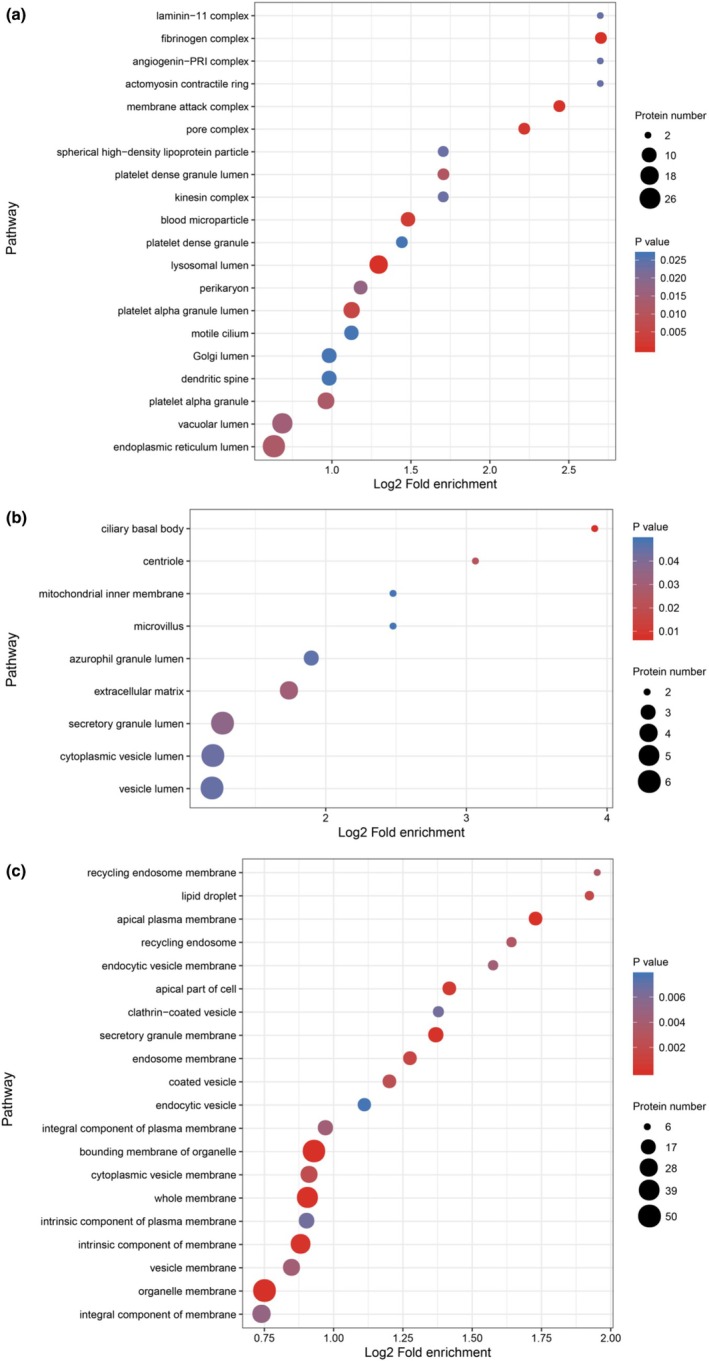
GO cellular component (CC) enrichment bubble plots of differentially expressed proteins. (a) Group C versus Group A; (b) Group C versus Group B; (c) Group A versus Group B. Bubble color indicates enrichment significance (*p* value), with a blue‐to‐red gradient; redder bubbles correspond to smaller *p* values and higher statistical significance.

**FIGURE 5:**
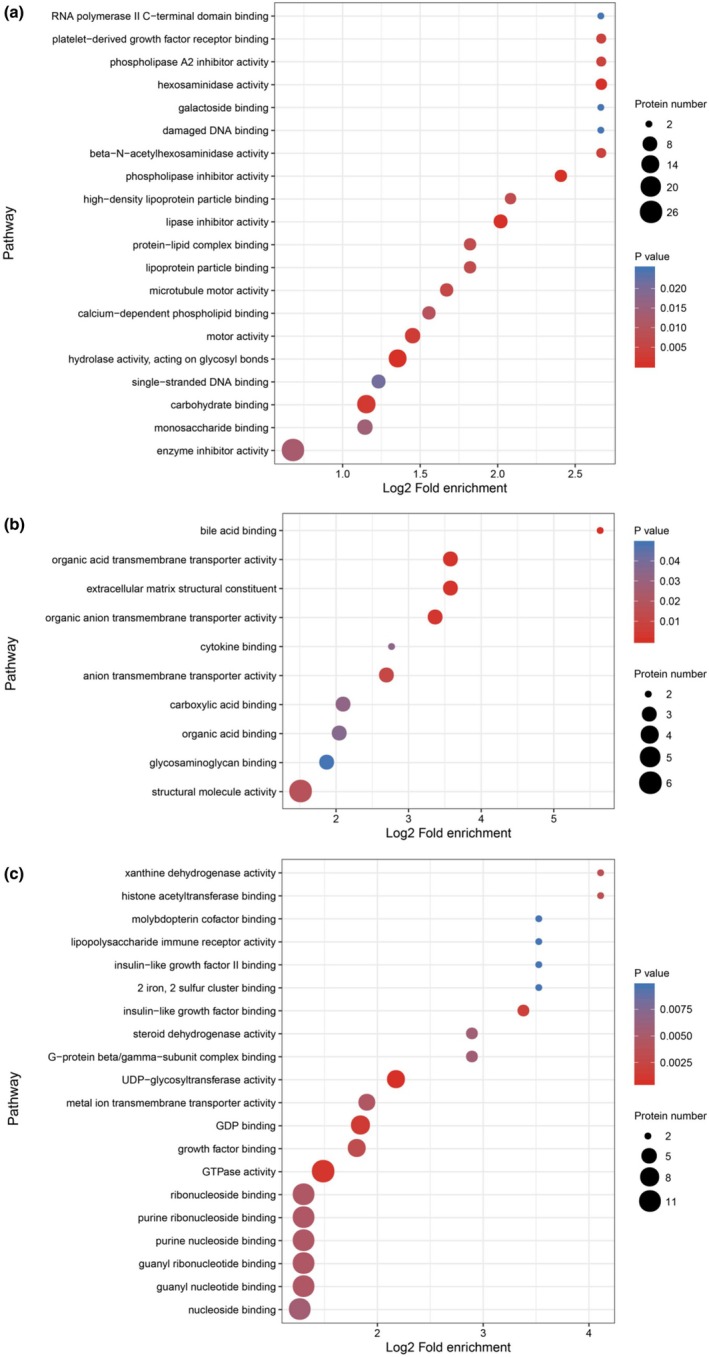
GO molecular function (MF) enrichment bubble plots of differentially expressed proteins. (a) Group C versus Group A; (b) Group C versus Group B; (c) Group A versus Group B. Bubble color indicates enrichment significance (*p* value), with a blue‐to‐red gradient; redder bubbles correspond to smaller *p* values and higher statistical significance.

Gene Ontology (GO) enrichment analysis was performed to explore the biological processes, cellular components, and molecular functions associated with the differentially expressed proteins. In the comparison between Group C and Group A, significant enrichment was observed in processes such as regulation of fibroblast proliferation, hemostasis, coagulation, and glycolipid metabolism (Figure 3). Cellular‐component analysis revealed enrichment in the extracellular matrix and cytoskeletal structures (Figure 4). Molecular functions, including protein binding and catalytic activity, were also highlighted (Figure 5). These findings indicate that the differentially expressed proteins participate in critical biological processes and molecular functions that may influence infant intestinal function.

As shown in Figure 3a, differentially expressed proteins between Group C and Group A (A) were significantly enriched in biological processes related to regulation of fibroblast proliferation, hemostasis, coagulation, and glycolipid metabolism. In contrast, fewer enrichment terms were observed in comparisons involving Group B (Figure 3b,c).

As shown in the cellular component enrichment analysis (Figure 4), the differentially expressed proteins between Group C (infrequent defecation) and Group A (normal defecation) were predominantly localized to the extracellular matrix (ECM) and cytoskeletal structures (Figure 4a). In contrast, comparisons involving Group B (frequent defecation) showed fewer and less significant enrichments for cellular component terms (Figure 4b,c).

The molecular function analysis (Figure 5) revealed that proteins differentially expressed in the Group C versus Group A comparison were significantly enriched in protein binding and catalytic activity (Figure 5a). This enrichment pattern points to widespread alterations in protein interaction networks and enzymatic functions in breast milk from mothers whose infants have infrequent defecation. Notably, these molecular function terms were not significantly enriched in the comparisons involving Group B (Figure 5b,c), reinforcing the unique proteomic signature associated with reduced stool frequency.

### 3.5 KEGG pathway enrichment analysis

**FIGURE 6:**
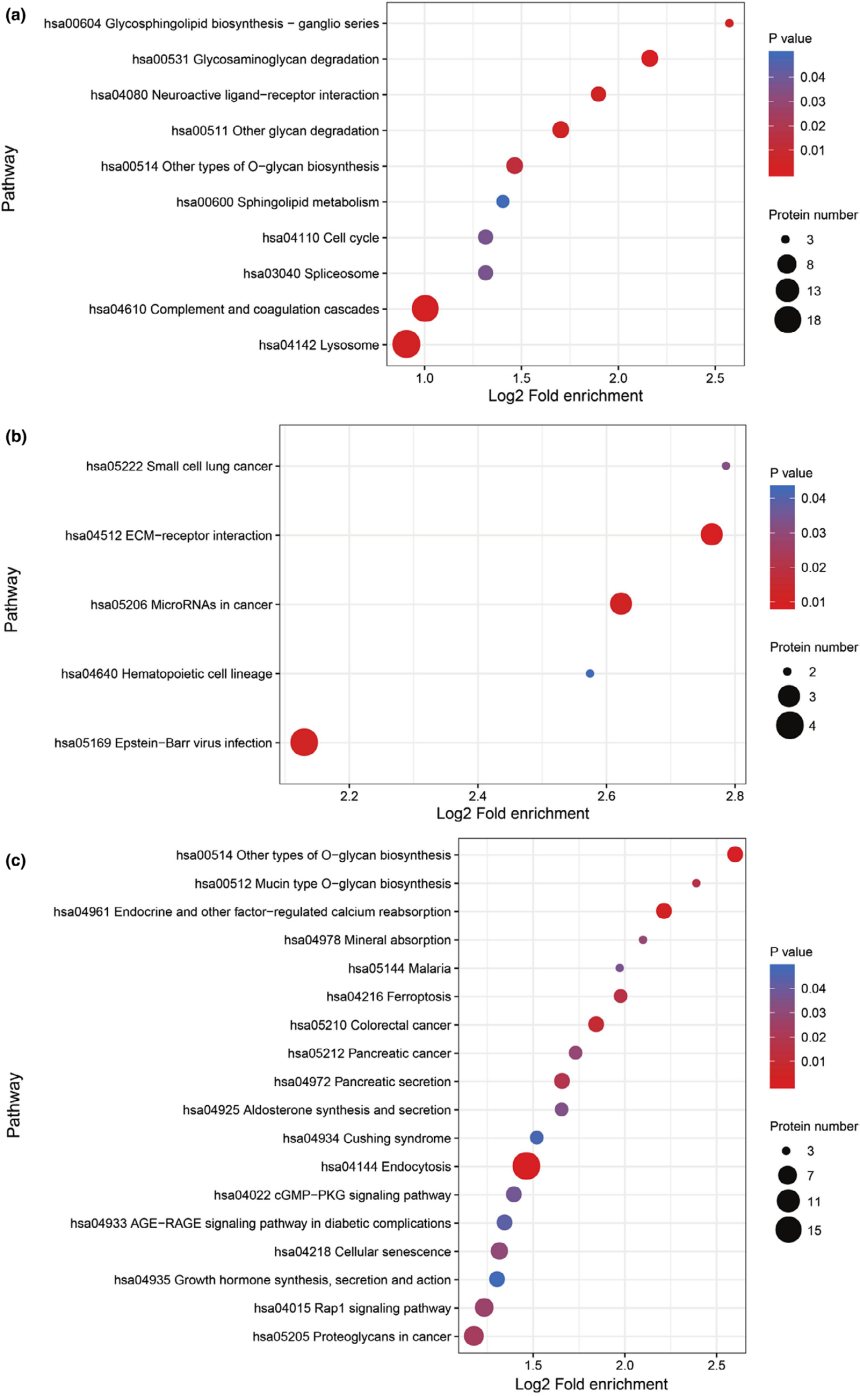
KEGG pathway enrichment analysis. Bubble plot showing KEGG pathway enrichment for differentially expressed proteins across Group C versus Group A (a), Group B versus. Group A (b), Group B and Group C (c). The bubble size represents the number of enriched proteins, and the color gradient (blue to red) reflects the *p* value of significance.

**FIGURE 7:**
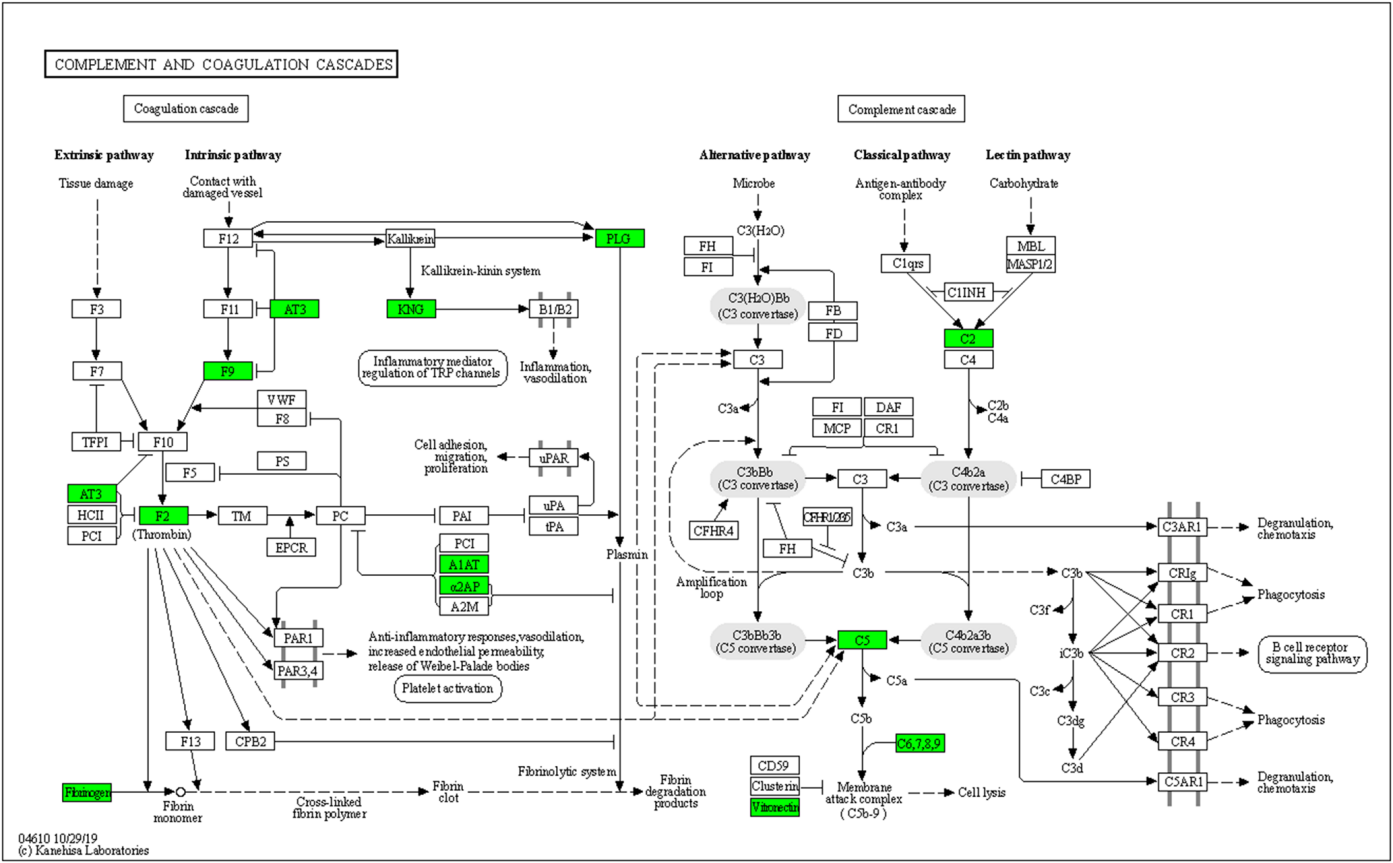
Complement and coagulation cascade pathway. KEEG pathway diagram showing the complement and coagulation cascade, which exhibited significant enrichment in the differential expression analysis between Group C and Group A. Proteins highlighted in the pathway indicate differential expression with statistical significance.

KEGG pathway analysis was performed to identify metabolic and signaling pathways significantly associated with the observed protein‐expression differences. In group C vs. group A, pathways such as glycosaminoglycan degradation, complement and coagulation cascades, and lysosome pathways were significantly enriched (Figure 6). Among these, the complement and coagulation cascade pathway showed the greatest enrichment (Figure 7). These findings suggest a mechanistic link between breast‐milk protein composition and infant defecation frequency, potentially mediated by metabolic and immune processes.

### 3.6 Protein–protein interaction (PPI) network analysis

See (Figure 8).

**FIGURE 8:**
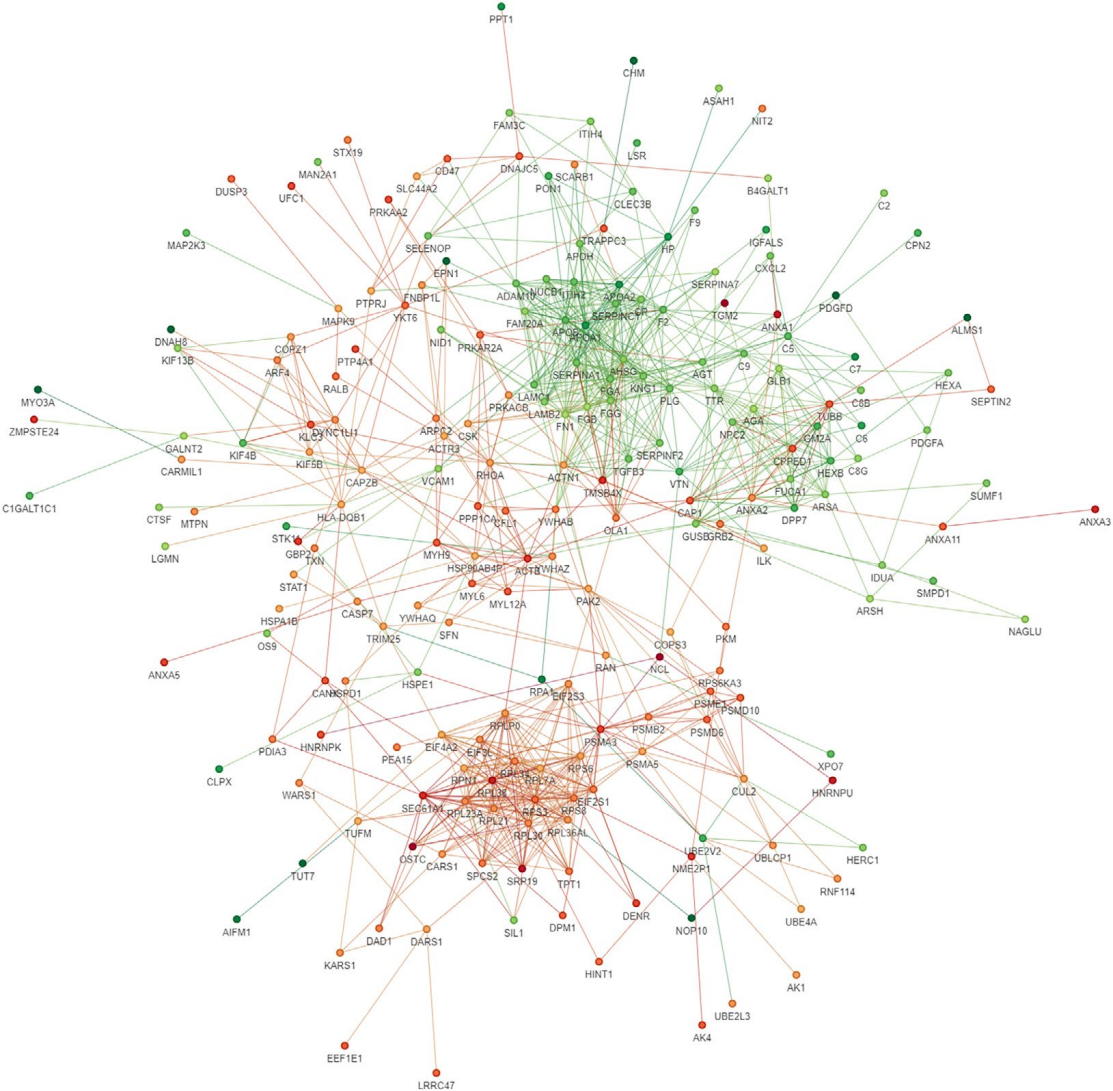
Protein–protein interaction (PPI) analysis was performed to identify key networks formed by the differentially expressed proteins. In the Group C versus Group A comparison, fibrinogen α‐chain (FGA) and fibrinogen β‐chain (FGB) emerged as central nodes in the interaction network. These interactions suggest coordinated roles in pathways related to intestinal function and metabolism. Overall, the PPI analysis highlights hub proteins that may regulate physiological processes influencing defecation frequency.

### 3.7 Casein protein analysis

As shown in Table 3, we identified significantly differentially expressed proteins between Group A and Group C. The key finding was a marked down‐regulation of α_s1_‐casein, β‐casein, and κ‐casein in Group C (Ratio values <0.6, indicating a 40% reduction relative to Group A; all *p* < 0.05). Notably, β‐casein exhibited the most pronounced down‐regulation (*p* = 0.0003). In contrast, α‐lactalbumin expression did not differ between the groups (Ratio ≈1, *p* > 0.05). Importantly, comparisons involving Group B (vs. Group A or C) showed no significant differences for any caseins (all ratios ≈0.7 or 1, *p* > 0.05). These findings strongly implicate casein depletion (particularly β‐casein) in breast milk as a key factor associated with decreased defecation frequency in infants.

**TABLE 3:** Analysis of differential protein expression between groups.

Protein accession number	Protein name	Gene name	C/A ratio	C/A	B/C ratio	B/C	B/A ratio	B/A
				*p* value		*p* Value		*p* value
P47710	α_s1_‐casein	CSN1S1	0.632	0.0171	1.156	0.5987	0.73	0.1775
P05814	β‐casein	CSN2	0.611	0.0003	1.187	0.5589	0.725	0.1557
P07498	κ‐casein	CSN3	0.74	0.0399	1.053	0.9609	0.779	0.2738
P00709	α‐lactalbumin	LALBA	0.977	0.9692	0.711	0.2952	0.695	0.3085

## 4 DISCUSSION

This study applies TMT proteomics to explore associations between human milk proteins and infant bowel patterns, a field in which TMT's multiplexing capacity is particularly valuable given the limited sample volumes available. The advantage of TMT technology in multiplex sample parallel quantification is particularly for efficient relative quantitative analysis of breast milk proteomics. In cases of limited sample size, the multiplexing capabilities of TMT technology offer significant advantages.

- simultaneous comparison of multiple samples through standardized labeling, which minimizes technical variability,
- high‐sensitivity detection of low‐abundance proteins (≥10 amol/μL) in complex breast‐milk matrices (Zecha et al., 2019), and
- comprehensive coverage of 2500+ proteins per run. Therefore, in the study of the association between breast milk composition and infant defecation frequency, the combination of TMT‐based proteomics and bioinformatics analysis methods can help reveal potential biomarkers and their mechanisms of action. As an exploratory pilot study with a limited sample size, the findings presented here are observational and correlational. While we discuss potential mechanistic interpretations based on existing literature, these should be considered as hypotheses for future research rather than conclusions directly supported by the current dataset.

Among the differentially expressed proteins, αs1‐casein, β‐casein, and κ‐casein were significantly down‐regulated in Group C compared with Group A. These caseins play vital roles in supplying essential amino acids and promoting the growth of beneficial gut microbes. For example, κ‐casein supports intestinal‐barrier integrity by facilitating early microbial colonization, whereas αs1‐casein contributes to gut mucosal immunity and digestion (Lalor & O'Neill, 2019; Mastromarino et al., 2014). β‐casein may be associated with the absorption of amino acids, peptides, and minerals, and may play a role in gut motility regulation. GO analysis revealed enrichment in processes such as hemostasis regulation, wound healing, and fibroblast proliferation, whereas KEGG analysis highlighted pathways including complement and coagulation cascades and glycosaminoglycan degradation. Together, these pathways suggest a mechanistic link between breast‐milk protein composition and infant intestinal function, potentially mediated by immune modulation and metabolic processes (Meng et al., 2023a) (Sadler & Smith, 2014; Yang et al., 2021).

The PPI network identified fibrinogen α‐ (FGA) and β‐ (FGB) chains as key nodes among the differentially expressed proteins in the comparison of Group C versus Group A. FGA and FGB are integral to coagulation and wound healing, processes that may indirectly influence gut motility and intestinal stability. Their strong connectivity within the network suggests interactions with other proteins involved in immune regulation and metabolic processes, thereby further affecting infant intestinal function. These findings are consistent with previous studies that highlight the role of fibrinogen‐related proteins in intestinal homeostasis (Brooke‐Taylor et al., 2017; Mastromarino et al., 2014).

β‐casein, the most significantly down‐regulated protein in Group C, may play an important role in intestinal health. Structurally, human β‐casein forms smaller, looser micelles than bovine β‐casein, facilitating easier digestion and nutrient absorption. These micelles promote the release of bioactive peptides that enhance the uptake of essential amino acids and minerals (Meng et al., 2023a; Sadler & Smith, 2014; Yang et al., 2021). According to previous studies, structural differences between A1 and A2 β‐casein variants lead to functionally distinct bioactive peptides during digestion: A1 β‐casein yields β‐casomorphin‐7 (BCM‐7), which suppresses gut motility in vitro (Brooke‐Taylor et al., 2017), whereas A2 β‐casein produces BCM‐9, a peptide associated with improved gastrointestinal function in clinical trials involving toddlers (Meng et al., 2023b).

Based on the functional differences among the variants mentioned above, the reduced β‐casein levels observed in Group C (infrequent defecation) may create a functional deficit analogous to the inhibitory effects of A1 β‐casein on intestinal motility. However, this hypothesized mechanism was not directly tested in the present study and requires experimental validation in future research. Building on evidence from a recent randomized controlled trial in Chinese toddlers showing that A2‐based growing‐up milk was associated with lower parent‐reported constipation scores (Meng et al., 2023a), future research should investigate whether A2 β‐casein supplementation can enhance gut motility in breastfed infants through well‐designed intervention studies that assess motility parameters, compare feeding regimens, and establish optimal dosing protocols for maximum therapeutic benefit.

Breast‐milk bioactive compounds, including β‐casein, significantly influence gut microbiota composition and intestinal health. β‐casein‐derived peptides exhibit antimicrobial activity, inhibiting pathogenic bacteria while supporting beneficial microbes such as Bifidobacterium (Brück et al., 2006; Dingess et al., 2021). Functional constipation has been linked to reduced short‐chain fatty‐acid (SCFA) levels and increased secondary bile acids, both of which are modulated by the gut microbiota. It is hypothesized that β‐casein's effects on the gut microbiota could influence SCFA production and gut motility. However, this proposed mechanism was not directly investigated in the present study and requires future validation (Lyons et al., 2020; Meng et al., 2023a).

Comparative analysis revealed significantly lower levels of both αs1‐casein and κ‐casein in Group C (reduced defecation) versus Group A (normal defecation) (*p* < 0.05). The mechanistic roles of these caseins involve distinct yet complementary functions:

1. κ‐casein acts as a mucosal‐receptor analog that competitively inhibits pathogenic bacterial adhesion (Lönnerdal, 2003); and
2. αs1‐casein (CSN1S1) exhibits potent immunomodulatory activity via p38 MAPK‐dependent stimulation of GM‐CSF and other pro‐inflammatory cytokines in monocytes (Vordenbaumen et al., 2011).

Based on the above research results, we tentatively propose a triple synergistic regulatory model of casein on the intestine: (i) κ‐casein–mediated control of microbial colonization, (ii) αs1‐casein–orchestrated immune modulation, and (iii) β‐casein–regulated nutrient absorption and motility (as discussed earlier). It should be emphasized that this triple synergistic model is speculative and requires further experimental validation. The integrated action of these casein fractions appears to maintain intestinal homeostasis through coordinated effects on three critical axes: (a) microbial ecosystem balance, (b) immune surveillance, and (c) epithelial barrier integrity, collectively shaping defecation frequency patterns in exclusively breastfed infants.

Enrichment of glycolipid‐metabolism pathways in Group C further suggests that β‐casein influences microbial metabolism, which in turn affects infant defecation frequency.

While this study provides novel insights, several limitations should be acknowledged. First, this study was designed as an exploratory pilot investigation aimed at generating hypotheses rather than validating conclusions. The small sample size (*N* = 9, three per group) limits statistical power and generalizability, a common challenge in early‐stage human milk research, given strict inclusion criteria (e.g., exclusive breastfeeding, stable stool frequency). Nevertheless, we applied stringent statistical correction (FDR‐adjusted *p* < 0.05) and reported effect sizes (fold changes) to enhance interpretability.

To address these limitations, future studies should: (1) Validate β‐casein expression in larger cohorts (*n* ≥ 50 per group) using targeted proteomics (e.g., SRM/MRM); (2) Implement longitudinal designs (0–24 months) to clarify if early β‐casein levels predict later stool frequency; (3) Integrate breast milk proteomics with microbiome and metabolomic profiling, such as fecal metagenomics and SCFA quantification, to validate the observed associations and elucidate the underlying biological mechanisms linking β‐casein, the gut microbiota, and infant stool frequency, using multivariate models (e.g., mediation analysis) to adjust for confounders.

Moreover, while our study identifies β‐casein as a potential candidate associated with infant stool frequency, the mechanistic links between β‐casein, gut microbiota composition, and defecation frequency remain unelucidated.

Future work should prioritize functional validation through in vitro (e.g., intestinal cell models to assess microbiota‐β‐casein metabolite interactions) and in vivo (e.g., β‐casein supplementation or knockout animal models with gut microbiota profiling) experiments. These approaches will help clarify how β‐casein may regulate gut motility via microbiota‐mediated pathways (e.g., short‐chain fatty acid production, bile acid metabolism) (Dingess et al., 2021; Meng et al., 2023a), thereby filling the current gap in understanding the tripartite relationship among β‐casein, the microbiota, and defecation frequency.

Translationally, these findings will inform (i) the development of A2 β‐casein–enriched supplements (validated by randomized controlled trials (RCTs) monitoring defecation frequency and SCFA profiles), and (ii) optimized infant formulas incorporating β‐casein derivatives (e.g., BCM‐9 peptides) to mimic breast milk's microbiota‐modulating effects. An integrated approach bridges molecular mechanisms and clinical applications, addressing both functional constipation and NEC prevention while aligning with Rome IV FGID frameworks (Geng et al., 2017).

## 5 CONCLUSION

This study explored the relationship between breast‐milk protein composition and infant defecation frequency using TMT‐based proteomics, identifying 2563 proteins. β‐Casein levels were significantly lower in infants with reduced defecation frequency, and functional analysis highlighted glycolipid metabolism and coagulation‐regulation pathways as key contributors to gut function. Protein–protein interaction analysis further identified fibrinogen‐related proteins as central network nodes. Collectively, the results suggest that β‐casein may support intestinal health, possibly through its unique micellar structure and microbiota‐modulating properties. These findings may provide a scientific foundation for developing β‐casein‐fortified human‐milk supplements or optimized infant formulas, particularly those enriched with A2 β‐casein–derived peptides such as BCM‐9 to alleviate functional constipation.

## AUTHOR CONTRIBUTIONS

**Zhan‐Hua Li:** Conceptualization; data curation; formal analysis; investigation; methodology; project administration; resources; software; supervision; validation. **Zhen‐Rong Xie:** Conceptualization; data curation; investigation; methodology; resources; supervision; validation. **Meng Li:** Conceptualization; data curation; methodology; validation; visualization. **Jing‐Jing Xiong:** Conceptualization; data curation; formal analysis; funding acquisition; methodology; project administration. **Mei Liu:** Formal analysis; resources; software; supervision. **Yong‐Kun Huang:** Conceptualization; data curation; formal analysis; investigation; methodology; resources; supervision.

## FUNDING INFORMATION

No funding was received for this study.

## CONFLICT OF INTEREST STATEMENT

The authors declare no conflicts of interest.

## ETHICS STATEMENT

The study was conducted in accordance with the Declaration of Helsinki and was approved by the First Affiliated Hospital of Kunming Medical University (Ethical Batch Number: 2020 Lunshen L No. 23).

## CONSENT

Written informed consent was obtained from the guardians of all infant participants.

## Supporting information


Table S1.

## Data Availability

The mass spectrometry proteomics data have been deposited to the ProteomeXchange Consortium (https://proteomecentral.proteomexchange.org) via the iProX partner repository with the dataset identifier PXD078188. During the peer review process, the data are accessible via a private reviewer link. Please refer to the cover letter for access instructions.
